# Still With Me? Assessing the Persisting Relationship to a Deceased Loved-One - Validation of the “Continuing Bonds Scale” in a German Population

**DOI:** 10.1177/00302228221076622

**Published:** 2022-03-18

**Authors:** Dora Hopf, Monika Eckstein, Beate Ditzen, Corina Aguilar-Raab

**Affiliations:** 1 Institute of Medical Psychology, 9144 Heidelberg University Hospital, Heidelberg, Germany; 2 Ruprecht-Karls University, Heidelberg, Germany

**Keywords:** Continuing Bonds Scale, prolonged grief disorder, attachment, posttraumatic personal growth, bereavement complicated grief

## Abstract

Continuing the bond (CB) to a deceased loved one plays a clinically significant role in grief. We validated the Continuing Bonds Scale (CBS) examining externalized CB (illusions and hallucinations) versus internalized CB (use of the deceased as a secure base) in relation to risk factors of complicated grief and bereavement-related adjustment. Data from 364 bereaved German participants on CBS, Inventory of Complicated Grief (ICG), and Posttraumatic Personal Growth Inventory (PPGI) entered an exploratory factor analysis. This yielded a two-factor-solution representing externalized and internalized CB (*KMO* = .89, *χ2* = 2100.5, *df* = 120). Both factors demonstrated high internal consistency (Cronbach's α = .87). ICG and PPGI highly correlated with externalized and internalized CB. Cause of death and feelings of responsibility were associated with externalized CB. In the future, the use of the CBS could help predict problems in grief processing and consequently implement early interventions.

## Introduction

### Theoretical Background

Losing someone you love through their death is one of the most stressful life-events, which is accompanied by intense psychological and physiological reactions in the bereaved. Those reactions involve crying, yearning, insecurity, aggression, depressive and (psycho-) somatic symptoms (Biondi & Picardi, 1996; Zisook & Shear, 2009), but also neuroendocrine (Hopf et al., 2020), immunological (Knowles et al., 2019), and cardiovascular changes (Fagundes et al., 2018). Suffering from the loss of a loved one may even increase mortality amongst survivors (Manzoli et al., 2007; Moon et al., 2011), highlighting the massive effects of this experience. The individual’s response to the loss can be placed on a continuum that goes from “normal” grief to prolonged, complicated grief (CG). Since grief is an extremely complex process and reactions to a loss are expressed in very different ways, the definition of “normal” grief remains highly individual. However, on the one hand, typical psychological reactions involve feelings of insecurity, anxiety, aggression and depressive and (psycho-) somatic symptoms (Biondi & Picardi, 1996; Kristensen et al., 2012; Stroebe et al., 2001). On the other hand, CG is characterized by longing for and preoccupation with the deceased, accompanied by emotional distress that persists beyond 6 months after the loss (Steinig & Kersting, 2015). CG symptomatology is found in up to 10–20% of the bereaved individuals (Shear & Shair, 2005; Steinig & Kersting, 2015) and has been shown to be associated with depression, hypertension and cardiac problems, work and social impairment, psychotropic drug use, and reduced quality of life (Boelen & Prigerson, 2007; Bonanno et al., 2007; Neria et al., 2007; Simon et al., 2007). In addition, bereaved individuals are at increased risk of suicide and suicidal behavior (Agerbo, 2005; Latham & Prigerson, 2004; Prigerson & Slimack, 1999; Stroebe et al., 2005, 2007).

The term CG does not represent an official diagnosis but, instead, comprises a larger category, with diagnostic disordered grief encompassing a smaller group. This disordered grief is called Prolonged Grief Disorder (PGD) or Persistent Complex Bereavement Disorder, which just recently have been added to the ICD-XI (WHO, 2018) and the Diagnostic Manual for Psychiatric Disorders (DSM-5).

Although the loss of a loved one seems a final event that requires the physical detachment of the bereaved from the attachment figure, it does not mean that the emotional or psychological relationship with that person immediately ends (Root & Exline, 2014). According to the Continuing Bonds Theory (Root & Exline, 2014), which was inspired by the attachment theory (Bowlby, 1980), people sense that the relationship to the deceased is continuing over their death, transforming, but not terminating. This so-called Continuous Bond (CB) can also be described as “the presence of an ongoing inner relationship with the deceased person by the bereaved individual” (Stroebe & Schut, 2005). This post-death relationship manifests itself through thoughts of the deceased, reminiscence about the deceased (Marwit & Klass, 1995), telling stories about the deceased (Nickman et al., 1998), dreaming of the deceased (Black et al., 2020), looking at photographs (Foster et al., 2011), keeping possessions of the deceased (Nickman et al., 1998), but also through the influence of the deceased character, lifestyle, beliefs on the own every-day life, sometimes culminating in an interactive communication like the engagement in a direct communication with the deceased (Foster et al., 2011; Nickman et al., 1998). Within research on causes and effects of CB, there has been an ongoing discussion about whether CB is a purely natural and adaptive process, or whether it also has maladaptive components which hinder the surviving individuals from integrating the loss into their life (Field, 2006b; Fraley & Shaver, 1999; Klass & Steffen, 2017; Stroebe et al., 2010). Although back in the 20s century researchers were convinced that CB is rather maladaptive and hinders healthy grieving, more recently, it has been proposed it may be important and adaptive to psychological well-being and grief resolution (Field, 2006a). CB is considered a grief-specific coping strategy, being a source of solace for the survivors. However, the extent to which CB is (mal-) adaptive seems to depend on specific dimensions such as the degree of proximity or the locus of the CB (Field, 2006a; 2006b; Field & Filanosky, 2009; Field et al., 2005). Psychological proximity is the degree to which people reminisce the deceased person (in memory). Those reminiscences may involve externalized components such as hallucinating about or having illusions of the deceased. For example, illusions entail the misperception of a stranger as the deceased because he or she has similar characteristics to the deceased or sounds that are mistaken for the deceased’s voice. Hallucinations may similarly involve the misconstruction of an internally driven source of information as emanating from an external source, when lying in bed at night (Field, 2006a). Internalized components, on the other hand, entail an ongoing connection with the deceased, thoughts of the deceased as a role model and the use of their mental representation as an internalized secure base and safe haven on the other hand. Externalized (ext.) CB is hypothesized to be indicative of unresolved loss, as it reveals the surviving individual’s inability to realize that the deceased person is dead. Ext. CB could hinder the integration of the loss into one’s life, the resolution of grief and, in a long-term, lead to chronic symptom burden and a greater risk of developing chronic diseases, or even higher mortality (Field & Filanosky, 2009). On the other hand, internalized (int.) CB expressions may serve to facilitate the integration of the loss in one’s own life and thus fostering the resolution of grief. More precisely, int. CB nurtures the positive development of the surviving individual by helping to overcome the loss reaction and strengthening their life experiences in a long-term.

Due to its high importance for individual grief processing and the psychological and physiological health of the surviving loved ones, it is important to measure the CB construct adequately and to study CB and its associated factors. In line with hypotheses of the adaptiveness of the subscales of CB, for example, ext. CB has been shown to be highly associated with complicated grief symptoms, demonstrating its link to unresolved loss (Field & Filanosky, 2009).

On the contrary, int. CB has been hypothesized to be associated with post-traumatic personal growth, meaning personality-strengthening reactions to this stressful life-event (Lipp & O'Brien, 2020; Scholtes & Browne, 2014; Tedeschi et al., 2017).

Personal growth takes place as the individual successfully addresses the challenges associated with the loss (e.g., managing every-day life issues that have been previously managed by the deceased, or re-orienting of personal goals and perspectives) and emerges with a revised sense of self in the world (Tedeschi & Calhoun, 2004).

Furthermore, participants who find meaning or peace in their loss, tend to have higher int. CB and lower ext. CB scores than those who do not find meaning or peace (Neimeyer et al., 2006). It has also been hypothesized that ext. CB scores are influenced by the suddenness of death and feelings of responsibility for the death (Field & Filanosky, 2009). For example, sudden deaths as well as feelings of responsibility for the death have been shown to be associated with higher ext. CB scores, showing that they may serve as risk factors for maladaptive grieving. On the other hand, relationship closeness to the deceased has been found to be positively correlated with both ext. and int. CB (Field & Filanosky, 2009).

Attachment style may also play a role in CB. Sudden deaths are associated with higher ext. CB scores, as well as feelings of responsibility for the death. Just recently, it has been hypothesized that people with insecure (high anxious or avoidant) attachment have more difficulties to adapt to the loss and thus show higher ext. CB scores. This hypothesis has only partly been confirmed (Field & Filanosky, 2009; Ho et al., 2013) and needs further investigation.

There is only one existing questionnaire measuring internalized and externalized CB – the Continuing Bonds scale (CBS). The CBS was first developed by Field and his colleagues and validated in different forms and widely used in English-speaking samples (Field & Filanosky, 2009; Field et al., 1999, 2003; Scholtes & Browne, 2014; Stroebe et al., 2012) as well as in one Italian sample (De Luca et al., 2016). There are several versions with different subscales, item numbers and response formats. Only the latest version of the CBS introduces the two subscales - ext. CB and int. CB - (Field & Filanosky, 2009). So far, there is neither a German version of the CBS nor another validated German questionnaire which measures CB components.

### Present Study

The aim of this study was to examine the psychometric properties (factorial structure, item characteristics, reliability, and validity) of the translated German version of the CB scale. To evaluate the scale’s construct validity, we assessed its relationship with risk factors for developing long-term problems in the adaptation to the loss (type of death, relationship to the deceased, feeling responsible for the death, feeling at peace with the loss, attachment style), relationship closeness, posttraumatic growth and complicated grief symptoms. We hypothesized, that a violent death, feeling responsible for the death, and not feeling at peace with the loss are all associated with higher externalized CB scores. This association should go in the opposite direction or not be found for the int. CB subscale. Furthermore, we assumed that the closeness of the relationship to the deceased is positively linked with internalized and externalized CB. Higher insecure-anxious attachment style should be positively associated with externalized CB, and negatively or not associated with internalized CB. In line with the theoretical considerations and the previously found results (Field & Filanosky, 2009; Tedeschi et al., 2017), we finally assumed that the int. CB subscale is more strongly linked to posttraumatic growth, whereas the ext. CB subscale has stronger associations with complicated grief symptoms.

## Materials and Methods

### Participants and Procedure

This study was approved by the Ethics Commission of the Medical Faculty of Heidelberg, Germany. The Participants were recruited between May 6^th^ 2020 and October 19^th^ 2020 from online grief portals, grief funeral homes, bereavement groups, and hospices. Inclusion criteria were the age of at least 18 years, speaking German fluently, and having lost a close attachment relationship (parent, spouse or partner, child, or close friend) through death. The online survey was conducted via the platform *soscisurvey.de* and participation was voluntary and completely anonymous.

A total of *N* = 557 individuals participated in our online assessment. We excluded everyone who did not consent to participate (*n* = 6), everyone under the age of 18 (*n* = 1), participants who had lost a pet (*n* = 2), and those who dropped out after the first page (*n* = 45), so that *N* = 503 participants remained. From those participants *n* = 364 answered the Continuing Bonds scale. Thus, our final sample consisted of *n* = 35 men (9.5%), *n* = 327 women (89.8%) and *n* = 2 people of other sex (.5%) at the age of 18 to 78 (*M* = 48.16, *SD* = 13.32). Most of the participants had lost a child (35.4%) or a parent (24.5%), lost someone due to acute disease (27.5%), with a mean time since death of 2–5 years. Demographic characteristics are displayed in Table 1.

**Table 1.** Demographic Characteristics of the Sample (*N* = 364).

	Categories	*n* (%)
Gender	Female	327 (89.8)
	Male	35 (9.5)
	Diverse	2 (0.5)
Education	Elementary school	12 (3.3)
	High school	134 (36.8)
	College	194 (53.3)
	Still at school/training	13 (3.6)
Deceased’s relation to the bereaved	Child	125 (34.3)
	Spouse/partner	67 (18.4)
	Sibling	45 (12.4)
	Parent	89 (24.5)
	Unborn child	4 (1.1)
	Close friend	11 (3)
	Something else	23 (6.3)
Time since death	0–3 months	21 (5.8)
	3–6 months	21 (5.8)
	6–9 months	13 (3.6)
	9–12 months	12 (3.6)
	1–2 years	44 (12.1)
	2–5 years	71 (19.5)
	5–10 years	83 (22.8)
	10–20 years	76 (20.9)
	>20 years	19 (5.2)
Cause of death	Acute disease	100 (27.5)
	Accident	78 (21.4)
	Chronic disease	59 (16.2)
	Natural (unexpected)	52 (14.3)
	Suicide	26 (7.1)
	Natural (expected)	15 (4.1)
	Pregnancy loss	13 (3.6)
	Murder	5 (1.4)
	Other cause	13 (3.6)
Cause of death (dichotomous)	Non-violent	239 (65.7)
	Violent	109 (29.9)
Feeling responsible for the death	No	262 (72)
	Yes	67 (18.4)

*Note.* percentages refer to the total sample (*N* = 364); percentages of missings range between 0.8% and 4.4%.

### Measures

#### Demographics and Characteristics of the Deceased

At the beginning of the survey, the following demographic characteristics were assessed: Age, gender, and educational level. Characteristics of the deceased person were: Relation to the bereaved and cause of death (acute disease versus chronic disease versus natural (unexpected) versus natural (expected) versus accident versus suicide versus murder versus other cause), which was later dichotomized (violent versus non-violent). Finally, feelings of responsibility for the death (one dichotomous item with the options *yes/no*), being in peace with the loss (one dichotomous item with the options *yes/no*), and relationship closeness were measured.

### Experience in Close Relationships

The German short version of the Experience in Close Relationships Questionnaire (ECR-RD-8) (Ehrenthal et al., 2021) was used to assess attachment style, more precisely attachment-related anxiety versus attachment-related avoidance. Participants had to assess their feelings regarding close relationships in general, using a 7-point Likert scale (1 – *strongly disagree* to 7 – *strongly agree*). The questionnaire shows good internal consistency in this sample (anxiety scale: α* =* .78; avoidance scale: α* =* .87). Subscales (attachment related anxiety vs. attachment-related avoidance) were calculated by computing the average of the relevant items.

### Continuing Bonds Scale

The CB scale (Field & Filanosky, 2009), which is a self-report measure, was used to assess the ongoing relationship to the deceased. The original questionnaire consists of 16 items (with two subscales) which can be answered on a 4-point Likert scale regarding the past month. The externalized CB subscale with 6 items measures hallucinations and illusions of the deceased, indicative of unresolved loss (e.g., item 15 “I imagined that the deceased might suddenly appear as though still alive.”) (Field & Filanosky, 2009). The internalized CB subscale entails 10 items, which include thoughts of the deceased as a role model and safe haven (e.g., item 1 “I thought about the positive influence of the deceased on who I am today.”). The factor analysis conducted by Field and Filanosky confirmed the two factors structure (externalized vs. internalized CB) with an internal consistency of α =.73 and α = .92 respectively (Field & Filanosky, 2009).

In this study, we decided to utilize a 5-point Likert scale (0 – *not at all* to 4 – *constantly*) instead of the original 4-point Scale (0–3), to provide the opportunity to choose a neutral category. It has been previously shown that an additional middle category enhances the reliability and validity of self-report scales (O’Muircheartaigh et al., 1999) and that people tend to systematically (and not randomly) choose one adjacent category over the other if there is no middle option (Krosnick et al., 2009).

In this version with 5 response categories, internalized CB scores range between 0 and 50; whereas externalized CB scores range between 0 and 30. To obtain the German version, two German native-speaker translated the items into German. Then, they were back-translated into English. The translated German version were reviewed by comparing the original CBS with the back-translated CBS by discussing and adjusting the items until reaching consent regarding the exact wording. The final version of the German CBS can be found in Appendix A.

### Inventory of Complicated Grief

In order to measure complicated grief symptoms, we used the German version of the Inventory of Complicated Grief (ICG-D) (Lumbeck et al., 2012). The ICG was originally developed to identify grief-related symptoms that could help discriminate between uncomplicated and complicated grievers (people reporting high levels of maladaptive aspects of grief) (Prigerson et al., 1995). Exemplary items are “I feel bitter over the person’s death”, or “I feel stunned or dazed about what happened”. The ICG consists of 19 items and the participants report the frequency with which they currently experienced each of the emotional, cognitive, and behavioral states on a 5-point Likert scale (0 *never* – 1 *rarely* – 2 *sometimes* – 3 *often* – 4 *always*). ICG-D sum scores range between 0 and 76. The one-factor-structure as well as reliability and validity of the ICG-D have been examined with good results (Lumbeck et al., 2012). Within the present sample, the ICG-D shows excellent internal consistency (Cronbach’s α= .90).

### Posttraumatic Personal Growth Inventory

We assessed posttraumatic growth via the Posttraumatic Growth Inventory (PTGI) (Tedeschi & Calhoun, 1996). The PTGI consists of 21 items and 5 subscales (“New possibilities”, “Personal strengths”, “appreciation of life” and “Religious changes”). Participants were asked to indicate the strength of changes that had been caused by the most stressful life event via a 6-point Likert Scale (*not at all – hardly – a little – quite – strong – very strong*). Therefore, PTPG total sum score ranges between 0 and 126. The questionnaire used in this survey was the translated and validated German version (Maercker & Langner, 2001) and has high overall internal consistency within this sample (Cronbachs α = .93), with subscale-specific consistencies between α = .78 and α = .92. Exemplary items are “I’m able to do better things with my life.“, “New opportunities are available which wouldn't have been otherwise.”

### Statistical Analysis

To evaluate the feasibility of the data for Exploratory Factor Analysis (EFA), we calculated Bartlett’s test of sphericity to value that the variables are correlated, and the Kaiser-Meyer-Olkin (KMO) test to measure sampling adequacy. Furthermore, to test for problematic multicollinearity between the variables, we calculated the determinant of the correlation matrix, which should be higher than .00001 (Field et al., 2012). We used the Screeplot with scree test and parallel analysis to assess the optimal number of factors, as recommended by Field (Field et al., 2012). Then we conducted a principal axis analysis (PAA) with oblique rotation (oblimin) on the set of 16 items, as recommended by Field and Filanosky (Field & Filanosky, 2009), and only items that loaded >.40 were retained (Thompson, 2004).

Furthermore, confirmatory factor analysis (CFA) was performed to assess the model fit (Tanaka et al., 1991). The item correlation matrix indicated that there is linearity in the variable pairs. Mardia test was calculated to test for multivariate normality (Mardia, 1974). Mardia test and QQ plots indicated non-normality of the data (mardia skewness: χ^2^ = 1835.724, *p* < .001; mardia kurtosis: χ^2^ = 12.584, *p* < .001). Therefore, we used the robust maximum likelihood method to estimate and interpret the robust standard errors. For the assessment of the model fit, the following fit indices were considered: Comparative Fit Index (CFI) (Bentler, 1990), Tucker Lewis Index (TLI) (Tucker & Lewis, 1973) and Root Mean Square Error of Approximation (RMSEA). Guidelines suggested that CFI and TLI equal to .90 or above (Bentler, 1990; Bollen, 1989), and RMSEA equal to .05 or below (Brown & Cudeck, 1993; Hu & Bentler, 1998) were indicative of a good fit. An internal consistency reliability analysis was performed for each factor using Cronbach’s alpha coefficients.

In preparation of the validity analyses, we calculated spearman rank correlations, *t-*tests and one-way analyses of variance (ANOVA) to detect if the demographic variables had any associations with the variables of interest (CB subscales, ICG-D scores, PTPG-scores and ECR-RD8 scores).

For validity analysis, we performed one-way analyses of covariance (ANCOVA) for the association between type of death, relationship to the deceased, feeling responsible for the death, as well as being at peace with the death (independent variables) and the CB subscales (dependent variables), including potential demographic characteristics (age, gender, time since death occurred) as covariates. For associations between CB subscales and attachment style, posttraumatic personal growth and complicated grief, we conducted partial correlations with potential demographic characteristics being ruled out.

Both EFA and CFA were performed with *R* version 3.0.3 (The *R* Foundation for Statistical Computing, Vienna, Austria). Item characteristics as well as all other analyses were performed using IBM® SPSS® Statistics for Windows version 27. The two-tailed significance level was set to *p* < .05.

## Results

### Exploratory Factor Analysis

**Figure 1.**
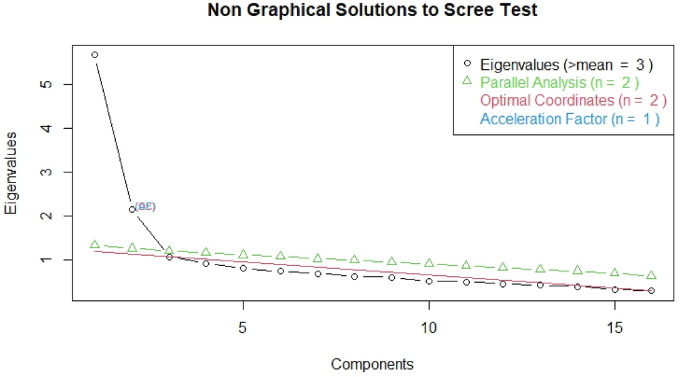
Results from the Scree Test prior to the Exploratory Factor Analysis.

The Kaiser–Meyer–Olkin measure verified the sampling adequacy for the analysis KMO = .89 (‘great’ according to Kaiser (1974)). All KMO values for individual items were >.83, which is well above the acceptable limit of .5. Bartlett’s test of sphericity (χ^2^ (120) = 2100.502, *p* < .001), indicated that correlations between items were sufficiently large for PAA. The scree plot showed inflexions that justify a 2-factor solution, which was also supported by the Parallel test (see Figure 1). Given the test and the scree plot as well as the theoretical and empirical considerations of the original publication, we decided to retain two components in the final analysis. Table 2 shows the factor loadings after rotation, as well as other item characteristics.

**Table 2.** Results From the Factor Analysis of the German Continuing Bonds Questionnaire (CBS).

	Factor Loadings						
Item No.	Int. CB^a^	Ext. CB^b^	Communalities	M (*SD*)	Item Difficulties	Item Discrimination	Cronbach’s α if tem is dropped
**1**	**.589**	−.161	.296	2.935 (*1.244*)	.587	.472	.872
**2**	**.687**	−.108	.424	2.025 (*1.383*)	.405	.583	.864
**3**	**.682**	−.087	.425	1.452 (*1.341*)	.29	.586	.864
**4**	**.578**	.182	.452	2.36 (*1.478*)	.472	.616	.862
**5**	**.767**	−.024	.574	1.854 (*1.423*)	.371	.699	.855
**6**	**.629**	.116	.469	1.854 (*1.34*9)	.371	.627	.861
**7**	**.687**	.003	.475	1.938 (*1.362*)	.388	.640	.860
**8**	**.569**	.083	.370	2.938 (*1.229*)	.588	.575	.865
**9**	**.548**	.137	.379	2.781 (*1.295*)	.556	.579	.865
**10**	**.499**	.317	.478	2.09 (*1.508*)	.418	.595	.864
**11**	.082	**.634**	.451	.882 (*1.31*)	.176	.547	.739
**12**	.120	**.523**	.339	1.219 (*1.413*)	.244	.523	.744
**13**	−.028	**.479**	.219	.963 (*1.362*)	.193	.426	.767
**14**	.097	**.660**	.497	1.076 (*1.464*)	.215	.590	.726
**15**	.099	**.504**	.304	1.938 (*1.591*)	.388	.477	.759
**16**	−.131	**.759**	.513	.669 (*1.257*)	.134	.6	.728
**Eigenvalues**	4.15	2.53					
**% Of variance explained**	26	16					
**Cronbach’s **α	.88	.78					

*Note.* Rotated factor solutions are displayed; loadings above .40 are written in bold letters.

a int. CB = Continuing Bonds Scale, internalized subscale;

b ext. CB = Continuing Bonds Scale, externalized subscale.

A two-factor solution accounted for 42% of the total variance: Int. CB (eigenvalue = 4.15; variance explained = 26%) and ext. CB (eigenvalue = 2.53; variance explained = 16%). The discriminative power of all items is medium to high and lies between .427–.699. The Cronbach’s α values are satisfying (overall CB: α = .87, internalized CB subscale: α = .88; externalized CB subscale: α = .78).

### Confirmatory Factor Analysis

**Figure 2.**
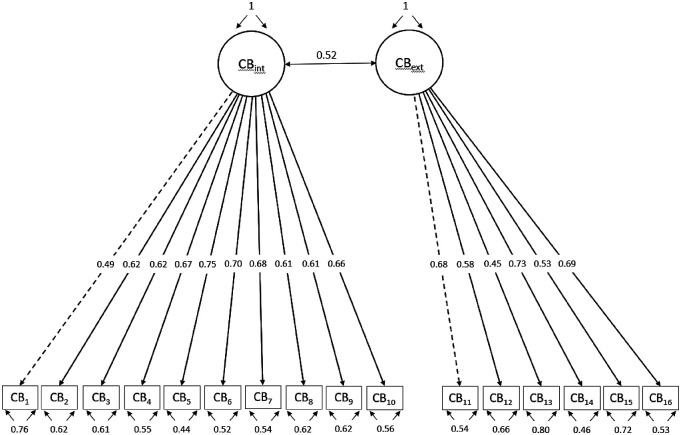
Standardized Factor Solutions from the Confirmatory Factor Analysis*.*

Confirmatory Factor analysis was conducted, with the CB items no. 1 to no. 10 representing factor1 and items no. 11 to no. 16 representing factor 2. Results indicated a fair fit of the two-factor model to the data (*TLI* = .84, *CFI* = .86, *RMSEA* = .08, *SRMR* = .07). Th path coefficients results of the CFA are displayed in Figure 2.

### Validity of the CB Scale

On average, participants had an int. CB score of *M* = 22.23 (*Range*: 0–69), and an ext. CB score of *M* = 6.72 (*Range*: 0–27). Furthermore, self-reported complicated grief symptoms were on average *M* = 32.12 (*Range*: 0–69), and PTPG total scores *M* = 53.61 (*Range*: 0–100). Descriptive statistics and intercorrelations between the variables of interest are shown in Tables 3 and 4, respectively.

**Table 3.** Means and Standard Deviations of the Main Outcomes of Interest.

	Int. CB	Ext. CB^b^
	M^a^ (*SD*)	M^c^ (*SD*)
**Type of death**		
Violent	22.13 (*9.01)*	7.71 (*5.45)*
Non-violent	22.27 (*9.5*)	5.93 (*5.59*)
**Relationship to the deceased**		
Child	21.46 (*9.62)*	7.72 (*6.21*)
Spouse/partner	22.68 (*9.74*)	7.77 (*6*)
Sibling	22.36 (*8.16)*	6.49 (*4.87*)
Parent	23.4 (*9.13)*	5.89 (*5.65*)
Unborn child	16.5 (*7.33)*	4.25 (*4.79*)
Close friend	21.82 (*10.29*	4.09 (*3.76)*
Something else	21.57 (*10.29*)	3.83 (*4.17*)
**CB total**	22.23 (*9.37*)	6.72 (*5.78*)
	M (SD)^d^	
**ICG** ^e^ **-D**	22.23 (*9.37*)	
**PTPG** ^f^ **total**	53.61 (*19.87*)	
PTPG appreciation of life	9.82 (*3.67*)	
PTPG new possibilities	12.31 (*5.73*)	
PTPG relationships with others	16.58 (*6.*32)	
PTPG personal strength	10.99 (*4.82*)	
PTPG spiritual change	3.96 (*3.38*)	

*Note.* This table presents means and standard deviations of int. CB, ext. CB, depending on type of death and relationship to the deceased, as well as means and standard deviations of the validity measures;

a int. CB = Continuing Bonds Scale, internalized subscale;

b ext. CB = Continuing Bonds Scale, externalized subscale;

c M = mean;

d SD = standard deviation;

e ICG-D = Inventory of Complicated Grief - German version;

f PTPG = Posttraumatic Personal Growth Inventory.

**Table 4.** Means, Standard Deviations, and Correlations of the Variables of Interest With Confidence Intervals.

Variable	*M*	*SD*	1	2	3	4	5	6	7	8	9	10	11
1. ECR	11.36	6.30											
Anxiety													
2. ECR	11.20	6.36	.25**										
Avoidance													
			[.14, .35]										
3. ICG	32.12	13.64	.13*	.17**									
			[.02, .24]	[.06, .27]									
4. CB	22.23	9.37	.17**	−.00	.37**								
Internalized													
			[.06, .27]	[−.12, .11]	[.27, .45]								
5. CB externalized	6.72	5.78	.05	−.01	.34**	.43**							
			[−.07, .16]	[−.12, .10]	[.25, .43]	[.34, .51]							
6. CB total	28.97	12.98	.14*	−.01	.42**	.92**	.76**						
			[.03, .25]	[−.12, .10]	[.33, .50]	[.90, .93]	[.71, .80]						
7. PTPG appreciation of life	9.82	3.67	−.05	−.08	−.14*	.07	.06	.07					
			[−.16, .06]	[−.19, .03]	[−.24, −.03]	[−.04, .17]	[−.05, .17]	[−.04, .18]					
8. PTPG new possibilities	12.31	5.73	−.04	−.10	−.20**	.10	.15**	.14*	.72**				
			[−.15, .07]	[−.21, .01]	[−.31, −.10]	[-.01, .20]	[.04, .25]	[.03, .24]	[.67, .77]				
9. PTPG relationship with others	16.58	6.32	−.06	−.27**	−.06	.19**	.16**	.21**	.62**	.66**			
			[−.17, .05]	[−.37, −.16]	[−.17, .05]	[.08, .29]	[.06, .27]	[.10, .31]	[.55, .68]	[.60, .72]			
10. PTPG personal strength	10.99	4.82	-.06	-.17**	-.21**	.12*	.16**	.15**	.70**	.79**	.63**		
			[-.17, .06]	[-.27, −.06]	[−.31, −.10]	[.01, .22]	[.05, .27]	[.05, .26]	[.64, .75]	[.74, .82]	[.55, .69]		
11. PTPG spiritual change	3.96	3.38	.04	−.07	−.03	.21**	.24**	.26**	.38**	.41**	.45**	.44**	
			[−.07, .15]	[−.18, .04]	[−.14, .08]	[.11, .31]	[.13, .34]	[.15, .36]	[.28, .47]	[.32, .50]	[.36, .53]	[.35, .52]	
12. PTPG total	53.61	19.87	-.05	−.18**	−.16**	.16**	.19**	.20**	.83**	.90**	.85**	.88**	.61**
			[−.16, .07]	[−.29, −.07]	[−.26, −.05]	[.06, .27]	[.08, .29]	[.09, .31]	[.79, .86]	[.87, .92]	[.82, .88]	[.85, .90]	[.54, .68]

*Note. M* and *SD* are used to represent mean and standard deviation, respectively. Values in square brackets indicate the 95% confidence interval for each correlation. The confidence interval is a plausible range of population correlations that could have caused the sample correlation (Cumming, 2014). * indicates *p* < .05. ** indicates *p* < .01.

Before conducting the validity analyses, we assessed whether control variables (age, gender, and time since death) correlated with our variables of interest (CB scores, ICG-D scores, and PTPG scores). Age significantly correlated with both the int. CB scale (*r* = - .11, *p* < .05) and the ext. CB scale (*r* = .13, *p* < .05). Gender did not correlate with either of the variables, except for the ICG-D scores: Complicated Grief Symptoms were significantly lower in men than in women (*t*(40.22) = - 2.48, *p* = .02, *Cohens d* = .486). Time since death had no associations with int. CB (*Spearman rho* = - .09; *p* = .12), but significant associations with ext. CB (*Spearman rho* = .13; *p* = .02), ICG-D scores (*Spearman rho* = - .13; *p* = .01), and PTPG scores (*Spearman rho* = .26; *p* < .001). We included all the significant variables as control variables into our models.

#### Cause of Death

To assess the association between cause of death (violent vs. non-violent) and ext. versus int. CB, we conducted two one-way ANOVAs with int. and ext. CB as dependent variables and type of death as independent variables. Age and time since death were included as covariates (time since death only for ext. CB). As expected, there was no significant association of death type with int. CB (*F*(1,337) = .01; *p* = .93), but a sig. association with ext. CB (*F*(1,336) = 6.29; *p* = .01; η^2^ = .02), showing that participants who lost someone due to a violent death showed significantly higher ext. CB scores than participants who lost someone due to a non-violent death.

#### Relationship to the Deceased

To assess the association between the relationship to the deceased and int. versus ext. CB, we conducted two ANCOVAs with the relationship as independent and the CB subscales as dependent variables. Age and time since death were included as covariates (time since death only for ext. CB). The results yielded no significant overall associations with the int. CB subscale (*F*(6,348) = .86; *p* = .52; partial η^2^ = .03) and no significant overall associations with the ext. CB subscale (*F*(6,347) = 1.96; *p* = .07; partial η^2^ = .001). Furthermore, linear contrasts were significant (estimated mean difference = - 3.7; *p* = .003), showing that the ext. CB scores were higher the closer the relationship to the deceased person was.

#### Feeling Responsible for the Death and CB

As expected, feeling responsible for the death had no significant association with int. CB (*F*(1,314) = 1.72; *p* = .19, partial η^2^ = .005), but a significant overall association with ext. CB (*F*(1,317) = 4.27; *p* = .04; partial η^2^ = .03). People who felt responsible for the death, showed significantly higher CB scores (*M* = 7.94; *SD* = .71) than those who did not feel responsible (*M* = 6.3; *SD* = .36).

#### Attachment Style and CB (Anxious Attachment Subscale)

Partial correlations indicated, that externalized CB did neither correlate with attachment-related anxiety (ECR subscale) (*r*_*p*_(302) = .06; *p* = .29), nor with attachment-related avoidance (*r*_*p*_(303) = - .02; *p* = .71), while controlling for age and time since death. However, both subscales correlate significantly positively with complicated grief (.13 and .17).

#### Feeling at Peace With the Loss and CB

To examine the association between feelings at peace with the loss and ext. versus int. CB, we conducted an ANOVA with Age and time since death as covariates (time since death only for ext. CB). There was no significant association between feeling at peace and ext. CB (*F*(1,274) = .34; *p* = .56, partial η^2^ = .001) and no significant association between feeling at peace and int. CB (*F*(1,273) = 3.03; *p* = .083; partial η^2^ = .011). People who found peace in the loss showed higher int. CB scores than people who did not find peace.

#### Associations Between CB and Complicated Grief

Because men and women differed in their level of complicated grief symptoms, we conducted partial correlations for men and women separately, with age (and time since death for the ext. Subscale) as control variables. Partial correlations between CG symptoms and ext. CB were highly significant (*r*_*p*_(317) = .35; *p* < .001) in women, but not significant in men (*r*_*p*_(32) = .31; *p* = .08). Partial correlations between CG symptoms and int. CB were highly significant for both women (*r*_*p*_(317) = .32; *p* < .001) and men (*r*_*p*_(317) = .58; *p* < .001).

#### Associations Between CB and Posttraumatic Personal Growth

Partial correlation analyses between internalized CB and posttraumatic personal growth subscales indicated significant small to medium correlations between CB and almost all of the PTPG subscales („Relationships with others”: *r*_*p*_ (311) = .19; *p* = .001; „Personal strength”: *r*_*p*_ (313) = .13; *p* = .02; Spiritual change”: *r*_*p*_ = .23, *p* < .001). Int. CB did not correlate significantly with the New possibilities“ subscale (*r*_*p*_ (313) = .1; *p* = .08), and not significantly with the „Appreciation of life” subscale (*r*_*p*_(313) = .07; *p* = .24). The overall PTPG scale correlation with int. CB was small but significant (*r*_*p*_ (314) = .17; *p* = .002). Partial Correlations analyses between externalized CB and posttraumatic personal growth subscales indicated small to medium correlations („Relationships with others“: *r*_*p*_ (313) = .16; *p* = .004; „Personal strength”: *r*_*p*_(315) = .15; *p* = .01; „Spiritual change”: *r*_*p*_ (315)= .22, *p* < .001, “New Possibilities”: *r*_*p*_ (315) = .114; *p* = .01). Ext. CB did not correlate significantly with the „Appreciation of life” subscale (*r*_*p*_ (315) = .06; *p* = .28). The overall PTPG scale correlation with ext. CB was small but significant (*r*_*p*_ (313) = .18; *p* = .001).

## Discussion

This study examined the validity of the German version of the two-factor CB Scale. Our validation study provides empirical evidence for a two-factor solution with 16 items. Hence, the CBS-G is a reliable instrument to measure internalized ongoing bond to the deceased and externalized components indicating aspects of unresolved loss. The CBS-G is time-saving and easily applicable in research and practice.

Based on the exploratory factor analysis suggesting a two-factorial solution as in the original English version, we tested the two-factorial solution by a confirmatory analysis, which yielded just barely satisfactory model fits. A single-factor solution did not yield significantly better fit indices either. Although a three-factor solution turns out statistically better than a two-factor solution according to the model fits, a two-factor solution makes much more sense for substantive reasons. We tested the distinction between the two subscales respectively and were able to present mostly sound evidence in support of it. Overall, the ability to internalize and stay connected to the deceased may be an adequate means to deal the experience of loss but could also manifest in more unfavorable ways.

Violent death, the closeness to the deceased and feeling responsible for the death may represent risk factors for an unfavorable trajectory toward complicated grief, so we tested the associations with ext. and int. CB: As expected, those had significantly higher ext. CB scores if they had lost someone violently, the closer they were to the deceased, and if they felt responsible for the death, which was not the case for int. CB, as hypothesized. On the one hand, this suggests the importance of these factors as risk- or, conversely, protective factors; on the other hand, it implies that unfavorable ext. CB processing is more likely while confronted with these unfavorable factors.

In contrast, higher int. CB expressions were found if bereaved individuals were able to make peace with the loss, although we were unable to uncover any significant correlations in this respect. Nevertheless, the peace-making dimension can be understood as a resource for the further mourning process, which is reflected, for instance, in the fact that the bereaved tend to have internalized a secure bond to the deceased.

Although the previous literature produced heterogeneous results, we expected to find a high correlation between ext. CB and the insecure-anxious attachment style. We assumed that the ability to internalize a secure attachment bond to the deceased should be rather impaired in the case of high levels of insecure-anxious attachment. However, contrary to prediction, we could not find any correlations between ext. CB with attachment style - neither with the anxiety nor the avoidance component. This could be due to several reasons, but we were not able to examine them within the scope of this study. Yet, our results here are consistent with those of the English validation study. One possible explanation could be that ext. CB, in terms of the difficulty of adequately integrating the experience of loss, does not express loss or separation per se, but rather the traumatic dimension of death. However, due in part to the majority of individuals in our sample who did not experience violent death, we cannot confirm this interpretation with our data. However, we were able to show a significant and positive correlation with complicated grief for both dimensions of attachment style. It is therefore reasonable to assume that attachment style plays an important role in the coping with a loss experience. Still, it remains to be clarified to what extent in under which circumstances the ability to maintain an inner bond with the deceased prevents the development towards pathology.

Interestingly, just about a third of the participants had lost a child – which is one of the most severe experiences of loss (d’Epinay et al., 2010), followed by a lost parent and finally a deceased partner in our sample. Why it was particularly individuals with the loss experience of a child who came forward in this difficult-to-recruit sample remains speculative. Although child-parent relations are particularly strong, it is still important to assess their relationship quality, as it may play a crucial role in how grief is experienced. On the one hand, low relationship quality may serve as a protective factor, while on the other hand, high relationship quality may be a risk factor for developing a maladaptive grief response.

We found that women differed significantly from the few men in our sample, in the additional burden of self-reported symptoms of complicated grief. There are already some studies that suggest gender differences in coping with loss experiences. For example, widows tend to have higher mean levels of traumatic grief, depressive and anxiety symptoms than widowers (Chen et al., 1999). When analyzing changes in prolonged grief symptoms across time, men seem to express prolonged grief as an acute, decreasing reaction, whereas women show an adjourned, mounting grief reaction (Lundorff et al., 2020). Furthermore, according to a recent meta-analysis, grieving adolescent girls tend to show higher levels of internalized grief responses and higher levels of PTSD symptoms than grieving boys (Shulla & Toomey, 2018). In general, differences between men and women in grief processing could also be mediated by psychobiological, historical, social, and cultural variables. Complex emotions such as guilt and shame vary between gender, probably due to traditional cultural roles of masculinity or femininity (De Boeck et al., 2018) and might also influence mourning behavior.

It is important to note that the concept of grief is perceived, processed and communicated differently depending on the culture we live in. For example, this can be seen in post-colonial African-American history, where grief and grief processing are described in much more melancholic terms similar to CB. Integrating cultural differences into research on grief and adapting self-report measures of grief to respective cultural habits provides a valuable expansion of our understanding of grief (see also Killikelly et al., 2020; Stelzer et al., 2020).

We found highly correlated ext. CB and CG symptoms only in women, but for women and men, comparatively strong associations between int. CB and CG symptoms. In the original validation study by Field and Filanosky, perceived closeness substantially contributed to these associations, and this may also differ between sexes (Field & Filanosky, 2009). In the future, especially longitudinal research should examine the direction and trajectory of grief, taking into account the degree of int. versus ext. CB. Additionally, to test measurement invariance in terms of gender and age will be important to verify in future studies.

Moreover, personal growth resulting from successfully overcoming the challenges associated with the loss can be understood as a resource. This therefore includes not only coping with everyday life and tasks, but also the reorientation of one’s own goal horizons and in relation to self-integrity. Surprisingly, we found not only significant, albeit partly small, correlations with int. CB, but also with ext. CB. Thus, we assume that both a more favorable integration of the loss is comparatively positively related to personal growth and a failed coping with the loss related to ext. CB. Differences in CB subscales do not necessarily translate into differences in everyday coping. There was no significant and positive association with the subscales of posttraumatic personal growth, reorientation or appreciation of life in either case. From these results we could conclude, that the ability to integrate the loss better or worse may be related to further variables that were not investigated in our study. For example, it is not captured in int. and ext. CB whether the affected person perceives the respective coping strategy positively or negatively in terms of relieving. Also, it could be that those who show highly ext. CB behaviors, etc., might have benefited from the severe adjustment period, especially if the death occurred a while ago. So, a temporal component could be important here and indicate to what extent one can personally grow from the event with increasing distance from the loss.

Overall, to establish a continuing bond towards a deceased close person seems to be an effective coping strategy. The differentiation of the various forms of CB could also make sense with regard to a temporal and developmental perspective: the immediate death of a close relative is usually difficult to comprehend and is sometimes accompanied by experiences comparable to shock reactions. An initial repression, which for example manifests itself in ext. CB. could, with a certain temporal distance to the loss, transform into int. CB. It would be predictively interesting to investigate to what extent the failure of the transition into an internal representation of the attachment figure is an expression of a lack of grief integration and leads to further unfavorable developments.

### Limitations

Our study has some limitations that are important to state. Its cross-sectional design does not allow interpretation of causality. Strong feelings of grief may lead to more intense CB, or vice versa. Against this background, it was also not possible to perform meditation analyses to shed light on mediating factors.

Our sample cannot be considered representative, as the majority of the mourners were female. This probably expresses a higher interest or also a higher willingness of female bereaved persons to consciously and proactively deal with these experiences. The sample showed some more important aspects worth mentioning: Overall, the majority of the sample was less burdened in relation to ext. CB, while also the values of self-reported symptoms of complicated grief as well as post-traumatic growth were in the medium range. Therefore, we did not base our analyses on an extremely burdened sample. This range restriction makes the sample less representative and may lead to less robust/more biased results.

This could be related, among other things, to the fact that for a quarter of the sample the time of death was between five and 10 years ago, and for another quarter it was as long as 20 years ago. Just about a third lost their relative due to an acute illness, followed by accidents and finally chronic illnesses. More than a half, consequently, did not lose the relative due to a violent cause, and the majority did not feel responsible for the death.

A last additional factor that remains open, but is nevertheless of considerable importance, is the qualitative experiential side of CB: Whoever loses someone by death may also get relief by expressing himself via ext. CB – in terms of avoiding the confrontation with the loss itself. However, how ext. and int. CB are predictive for the further course of an integration of the loss experience can only be clarified in a longitudinal design under consideration of further influencing factors but focusing on the emotional dimension. The differential predictive nature of the two subscales therefore needs further investigation.

### Implications/Strength

This is the first study that measures different types of ongoing attachment towards the deceased – ext. and int. CB – in a German population, using a newly translated questionnaire. The validation of the German CBS gives us the opportunity to use this self-report instrument in future research on predictors for both positive and negative grief-related mental health outcomes. With this investigation, we were able to extract both structural and relationship-related characteristics influencing the ongoing attachment to the deceased. Furthermore, as potential differences between ext. and int. CB in predicting posttraumatic growth as well as complicated grief symptoms could not be clearly extracted within our data, future investigations should examine potential personality-related, cultural social and historical factors influencing or moderating the ongoing attachment to the deceased loved one.

Regardless, intuitively, most people process the death of a near and dear one through a perpetuation of the internalized relational experience. The different configuration – in terms of int. and ext. CB – can thereby be more or less conducive to the development of complicated grief or other health-related strains. The use of this questionnaire could provide insights into the quality of grief processing in the bereaved and help to predict unresolved loss such as complicated grief. This may help to initiate early counseling when needed and, thereby, prevent prolonged burden.

## APPENDIX A

Continuing Bonds Scale German

Male Version

1. Ich habe über den positiven Einfluss des Verstorbenen auf meine heutige Person nachgedacht.

2. Ich war mir dessen bewusst, dass ich versuche mein Leben auf die Art zu leben, wie es der Verstorbene gewollt hätte.

3. Ich habe den Verstorbenen als Vorbild wahrgenommen, und möchte so sein wie er.

4. Ich habe mir vorgestellt, wie der Verstorbene mich leitet oder über mich wacht als ob sie unsichtbar aber anwesend wäre.

5. Wenn ich wichtige Entscheidungen treffen musste, habe ich überlegt, was der Verstorbene vielleicht gemacht hätte, und das als Hilfe für meine eigene Entscheidungsfindung genutzt.

6. Ich war mir dessen bewusst, dass ich versuche die Wünsche des Verstorbenen zu erfüllen.

7. Ich erlebte, wie der Verstorbene durch seinen Einfluss auf meine heutige Person weiterlebt.

8. Ich habe darüber nachgedacht, wie der Verstorbene etwas, das ich selbst gesehen oder gemacht habe, genossen hätte.

9. Ich habe mir vorgestellt, wie ich etwas Besonderes mit dem Verstorbenen teile, das mir passiert ist.

10. Ich habe mir vorgestellt, wie mich die Stimme des Verstorbenen ermuntert, durchzuhalten.

11. Ich habe tatsächlich gehört, wie die Stimme des Verstorbenen zu mir spricht.

12. Ich habe kurzzeitig gehandelt, als würde der Verstorbene noch leben – ich habe zum Beispiel seinen Namen gerufen, oder den Tisch für zwei gedeckt.

13. Ich habe andere Menschen mit dem Verstorbenen verwechselt, auch wenn nur für einen kurzen Moment

14. Ich habe die körperliche Berührung des Verstorbenen tatsächlich gespürt.

15. Ich habe mir vorgestellt, der Verstorbene könnte plötzlich erscheinen, als sei er noch am Leben.

16. Ich habe den Verstorbenen tatsächlich vor mir stehen sehen.

Items 1–10: internalized Continuing Bonds (CB int)
Items11–16: externalized Continuing Bonds (CB ext)

Scale goes from from 0 (“trifft überhaupt nicht zu”) to 4 (“trifft voll und ganz zu”) with respect to the last month

Female Version

1. Ich habe über den positiven Einfluss der Verstorbenen auf meine heutige Person nachgedacht.

2. Ich war mir dessen bewusst, dass ich versuche mein Leben auf die Art zu leben, wie es der Verstorbene gewollt hätte.

3. Ich habe den Verstorbenen als Vorbild wahrgenommen, und möchte so sein wie sie.

4. Ich habe mir vorgestellt, wie die Verstorbene mich leitet oder über mich wacht als ob sie unsichtbar aber anwesend wäre.

5. Wenn ich wichtige Entscheidungen treffen musste, habe ich überlegt, was die Verstorbene vielleicht gemacht hätte, und das als Hilfe für meine eigene Entscheidungsfindung genutzt.

6. Ich war mir dessen bewusst, dass ich versuche die Wünsche der Verstorbenen zu erfüllen.

7. Ich erlebte, wie die Verstorbene durch ihren Einfluss auf meine heutige Person weiterlebt.

8. Ich habe darüber nachgedacht, wie die Verstorbene etwas, das ich selbst gesehen oder gemacht habe, genossen hätte

9. Ich habe mir vorgestellt, wie ich etwas Besonderes mit der Verstorbenen teile, das mir passiert ist

10. Ich habe mir vorgestellt, wie mich die Stimme der Verstorbenen ermuntert, durchzuhalten.

11. Ich habe tatsächlich gehört, wie die Stimme der Verstorbenen zu mir spricht

12. Ich habe kurzzeitig gehandelt, als würde die Verstorbene noch leben – ich habe zum Beispiel seinen Namen gerufen, oder den Tisch für zwei gedeckt.

13. Ich habe andere Menschen mit der Verstorbenen verwechselt, auch wenn nur für einen kurzen Moment.

14. Ich habe die körperliche Berührung der Verstorbenen tatsächlich gespürt.

15. Ich habe mir vorgestellt, die Verstorbene könnte plötzlich erscheinen, als sei er noch am Leben.

16. Ich habe die Verstorbene tatsächlich vor mir stehen sehen.

Items 1–10: Internalized Continuing Bonds (CB int).

Items 11–16: Externalized Continuing Bonds (CB ext).

Scale goes from from 0 (“trifft überhaupt nicht zu”) to 4 (“trifft voll und ganz zu”) with respect to the last month.
